# The Role of the Orbitofrontal and Dorsolateral Prefrontal Cortices in Aesthetic Preference for Art

**DOI:** 10.3390/bs7020031

**Published:** 2017-05-11

**Authors:** Luca F. Ticini

**Affiliations:** Division of Neuroscience and Experimental Psychology, Faculty of Biological, Medical and Health Sciences, The University of Manchester, Manchester M13 9PL, UK; luca.ticini@manchester.ac.uk

**Keywords:** neuroaesthetics, preference, art, dorsolateral prefrontal cortex, orbitofrontal cortex, brain stimulation, tDCS

## Abstract

The search for the underlying neural activation that occurs during subjective aesthetic experiences of artwork has been enhanced through neuroimaging techniques. Recently, the dorsolateral prefrontal cortex, alongside the orbitofrontal cortex, have been implicated in aesthetic appreciation, and this is the focus of the present paper. Here, the validity of this conclusion is examined through the discussion of its neuroanatomical connections and functional properties. It is proposed that the experimental evidence challenges the view that this area could hold a privileged position in a brain network involved in aesthetic preference.

## 1. Background

In recent years, neuroimaging studies have investigated the underlying neural foundations of aesthetic experience of art (reviewed in [1,2]). A sizeable number of neuroimaging investigations demonstrated that the activity of the prefrontal cortex (PFC) reliably predicts subjective aesthetic experience (see Table S1 in [3]). The general consensus is that the medial orbitofrontal cortex (OFC; [4,5,6,7]) and particularly its subdivision A1 [6] is maximally engaged during the perception of beautiful stimuli, regardless of whether they are visual, musical or mathematical (e.g., [8]). A separate area appears equally prominent in mediating visual aesthetics for artwork, namely the left dorsolateral prefrontal cortex (DLPFC). The validity of this claim, originally based on electrophysiological investigations [9], was strengthened by two recent experiments in which the stimulation or the inhibition of this portion of the cortex increased [10] or decreased [11] preference ratings, respectively. In detail, the first work [10] showed that increasing the DLPFC’s excitability by means of transcranial direct current stimulation (tDCS) resulted in a very modest (3%) but significant increase of the preference ratings for representational paintings and photographs, but not for abstract art (for other findings on the left frontal activation in representational artwork, see [12,13]). The second investigation [11] showed that disruptive transcranial magnetic stimulation (TMS) selectively reduced how much participants liked visual art. Interestingly, in the latter experiment, representational and abstract paintings were selectively less liked in the group of participants that generally preferred figurative and abstract stimuli, respectively. Based on these results, the authors concluded that this prefrontal segment has a determinant role in the network shaping aesthetic preference. These findings are so interesting that I thought to critically investigate the assumption that the left DLPFC is a neurobiological correlate of the aesthetic experience. In this essay, I will outline the anatomical connections and functional properties of the DLPFC and will come to the conclusion that, rather than playing “a fundamental role in mediating esthetic appreciation” [11], its involvement has been overestimated.

## 2. The Prefrontal Cortex and Art Aesthetics

That said, identifying the contribution of each PFC area is recognised to be an arduous challenge (for review, see [14]). As a matter of fact, the PFC belongs to a complex neural network with heterogeneity of connections and functions (for a review [15]), in which other components participate in reward and affective states. Researchers working on aesthetics have been generally graceful in acknowledging this fact by not declaring that a particular area is the exclusive neural correlates of aesthetic preference [13]. Even so, functional magnetic resonance imaging (fMRI) studies have consistently isolated a parametric modulation of the OFC associated with beauty (e.g., [4,6]). This modulation is independent from the physical properties of the stimulus, a result that has already been known from the literature on reward processing (cf. [16,17]). Also, magnetoencephalography (MEG) revealed activity in the orbitofrontal sector of the PFC in response to artistic stimuli and pictures [9]. However, a parametric relationship with the aesthetic reports was not detected because the OFC’s anatomical position makes it unlikely to appear in scalp-recorded brain electrical signals (on the contrary, the DLPFC is easily accessible to scalp measurements). 

What is the known role of the OFC and of the DLPFC beyond the aesthetic domain? Neuroimaging (e.g., [16]) and lesions studies (e.g., [18]) associated the medial OFC with the representation of the reward value of an incoming stimulus. In addition, the incorporation of affective-related information dispatched by the interconnected limbic areas [19], such as the amygdala, is thought to signal to the OFC the value of the reward of a stimulus [20,21]. On the other hand, evidence gathered from DLPFC lesion studies in both humans [22] and non-human [23,24] primates determined that it has a small impact on tasks that require reward assessment [22,23]. This apparent functional distinction is also reflected in the anatomical position of these areas. As the OFC receives input from sensory pathways involved in object processing [25] and the inferior visual temporal cortex [26], it is in a favourable location to code for the reward value of the stimuli [22,23,24,27,28,29,30,31,32,33,34,35,36]. Unlike the OFC, the DLPFC is not directly connected to the primary sensory cortices [37,38], but has extensive communication with the posterior cortical regions (such as the parietal areas processing spatial information; [39]) and with motor areas in the frontal lobe [40]. Thus, it is ideally located to regulate the activity of other brain structures and, as such, it is involved in cognitive functions such as working memory, attention, response selection (e.g., [27,31,33]) and emotion regulation [14,41,42]. 

## 3. Physiological Investigations of the Pre-Frontal Cortex in Humans and Non-Human Primates

Additional insights divorcing the role of the OFC from that of the DLPFC were provided by Wallis and Miller [43] in a revealing neurophysiological investigation in non-human primates. These researchers simultaneously measured the activity of these two prefrontal structures while rhesus macaques choose between pictures associated with different quantities of reward (juice). In keeping with human fMRI studies (e.g., [44]) the authors found that, although both structures encoded the amount of reward, OFC neurons did so independently of the ensuing behavioural response. Instead, the DLPFC’s activity reflected how the animal used the reward information to guide its behaviour. In the context of our discussion, this result may question whether or not the DLPFC has a causal role in aesthetic preference and whether, during an aesthetic preference task, it is more involved in approach-related and goal-directed action planning ([45]; see also [46]). Already a decade ago, Chatterjee [47] anticipated the DLPFC’s possible involvement in aesthetic preference when a decision based on perceptual and affective information has to be taken. In humans, compelling experimental evidence of that was provided by Vanderhasselt and colleagues [48], among others. They demonstrated that DLPFC stimulation enhances cognitive control for positive affective stimuli, which is the capacity to modify one own’s behaviour to achieve a defined goal [49]. One year later, Davidson [14] expressed the view that differences in the experimental task could drive brain responses: “tasks that include a response component will be more likely to show affect-related PFC activation asymmetry in the dorsolateral regions and it is activity in these regions that are most likely to be reflected in scalp-recorded brain electrical signals”. The data confirm this hypothesis; activity in the left DLPFC is triggered when participants are asked to express their preference by pressing a button during stimuli presentation (such as in [5,9,10]). On the contrary, the orbitofrontal section is involved during passive perception of pre-selected art stimuli (i.e., rated before the experiment took place [4]; or after stimulus presentation [6]; for a possible association between mnemonic processes and aesthetic preference see [50,51]). For instance, in their fMRI experiment showing OFC activity, Kawabata and Zeki [4] first requested participants to select from a large pool of paintings, the paintings to be viewed by the subjects while performing in the fMRI study. In this phase, they rated each painting (abstract, still life, landscape, or portrait) on a scale from 1 to 10. Then, only the paintings pre-classified as ugly (1–2), beautiful (9–10) and neutral (5–6) were *passively* observed on a computer monitor in the scanner. In other two fMRI experiments highlighting OFC’s role in aesthetic preference, participants were asked to rate visual or auditory stimuli (as “beautiful”, “indifferent”, or “ugly” [6]) or to judge which of two paintings was more beautiful [7] by pressing predetermined buttons following each stimulus presentation. A different series of studies by Cela-Conde and colleagues [9] asked participants to respond during stimulus presentation. In their MEG study, one group of participants was instructed to raise a finger when they considered a picture to be beautiful and another group to raise the same finger when they considered the image not beautiful. They found a role of the DLPFC in the aesthetic judgment. In this experiment, as well as in the work by Cattaneo et al. [10,11], participants expressed their judgment whilst the image remained visible on the screen. In particular, in the tDCS paper [10], the participants indicated their response through a mouse click on a scale representing the level of liking, while in the TMS work [11] they performed key presses as fast as possible to indicate whether they liked the painting or not.

Did the experimental design affect the outcome of these experiments? Possibly, but this is not wholly clear yet, as the paradigms that have been used vary considerably. Importantly, a meta-analysis [52] revealed no significant differences in brain activation between studies in which subjective pleasantness judgements (attractiveness, liking or beauty) were reported during or after scanning. On the contrary, Ishizu and Zeki [7] demonstrated that the DLPFC is activated in perceptual (brightness judgement) as well as aesthetic judgement tasks, and that the OFC is only activated during the latter task. This again suggests a general involvement of the DLPFC which is not necessarily related to aesthetic preference. However, as this view contrasts with the results of Cattaneo and colleagues [10], in which tDCS of left DLPFC did not affect the control task in which the participants were requested to make colour judgements (“How colourful is this image?”), the response to this question requires further investigation.

Wallis and Miller [43] also demonstrated a difference in the time course of the activation of the OFC and the DLPFC. They measured common activity in both areas at around 300 ms, whilst a peak selectivity associated with reward information appeared earlier in the OFC (510 ms, mean value) than in the DLPFC (592 ms). For the authors, this indicated a direction of the flux of reward information, which would enter the PFC via the orbital area to be subsequently forwarded to the DLPFC in order to guide the forthcoming behavioural response [45]. This is in keeping with the human MEG study by Cela-Conde and colleagues [9], in which the modulation of the DLPFC relative to beautiful stimuli was shown in late latencies (400–900 ms) and not in early ones (100–300 ms). Overall, this may indicate that, also in aesthetic tasks, the left DLPFC engages in a subset of delayed computations used to control behaviour (e.g., plan an approach through goal-directed actions [45]) and that its activity is influenced by reward and other affective information passed on by the OFC. 

Another issue worth commenting is the left lateralisation of the DLPFC’s activity in aesthetic tasks (see also the paragraphs above). The left DLPFC is considered to be part of a neural network of mood modulation [53]. For instance, Davidson suggested that this electrophysiological asymmetry is associated with self-reported measures of mood, dispositional affect and behavioural inhibition [14]. As a matter of fact, anodal tDCS of the left DLPFC induces mood improvements and it is used in the treatment of major depression disorders [54]. Also, the offset of negative stimuli [55] prompts activity in the left DLPFC, perhaps indicating the DLPFC’s role in coping with negative events or, in other words, in cognitively reframing an affective state by regulating the activity of other areas, such as the amygdala [41]. 

Thus, is the behavioural effects measured by Cattaneo and colleagues [10] a result of an increased participants’ mood, rather than of their aesthetic preference? The results of their control task (colour judgements) would suggest that this is not the case. However, the idea that mood alters stimuli evaluation was discussed elsewhere [56], but it fits well with the “Affect Infusion Model” of Forgas [57] whereby a positive mood at the beginning of an aesthetic experience affects the quality of aesthetic processing. By the same token, Cupchik and colleagues observed left DLPFC activity when subjects were instructed to “approach the paintings in a subjective and engaged manner, experiencing the mood of the work and the feelings it evokes” [58]. Incidentally, individuals with higher left PFC activity also show lower levels of the stress hormone cortisol [59], they recover more rapidly from negative events [55] and report higher levels of psychological well-being [60]. 

## 4. Differences among Neuro-Recording Techniques

The last issue discussed here concerns whether or not tDCS, the technique employed by Cattaneo and colleagues [10,11], can demonstrate a causal role of the left DLPFC in the network of aesthetic preference. Here, I will suggest that the lack of incontrovertible knowledge about the local and remote effects of tDCS on the left DLPFC, and about the direction and pathway of tDCS currents beyond the area under the electrodes ([61,62,63], makes it difficult to draw firm conclusions. tDCS applies a constant electric current to the brain via two large electrodes (anodal and cathodal) placed on the scalp of an individual [64,65]. Along with other noninvasive neuromodulatory tools, it is widely used to complement traditional therapies in various neurological and psychiatric conditions such as stroke rehabilitation, pain and depression ([66,67,68]. Furthermore, when applied over the DLPFC, tDCS has various effects ranging from affecting decision-making, working memory, depression, and pain perception ([69,70,71,72,73]; for a review, see [74]) to decreasing the ratings of unpleasantness, discomfort or pain [71]. In spite of that, the mechanisms underlying the behavioural effects of tDCS are not completely understood [75]. Indeed, tDCS behavioural outcomes depend on a variety of factors, such as the position and polarity of the electrodes or their size, current intensity, density and duration of the stimulation, and the properties of the tissue in the targeted area (e.g., the sulcal anatomy, cf. [76]). At a local level, anodal and cathodal tDCS respectively increase and decrease the neuronal excitability by modifying the permeability of the cellular membranes to ions and molecules [65,77,78]. Although these polarity-specific local effects are assumed to be similar during and after stimulation [65,77,79], the underneath biophysics differs: tDCS affects membranes’ polarity locally during stimulation whilst it modulates synaptic neurotransmission after stimulation ([49,63,80]. Also, stimulation induces blood flow changes in regions anatomically connected to the targeted area, whilst a widespread decrease in cortical perfusion [81] accompanied by an increase in resting functional connectivity [82] is often detected after stimulation. This, added to the heterogeneity of anodal-excitation and cathodal-inhibition effects in cognitive studies (e.g., [83]), puts constraints on interpretations of such studies.

It is nonetheless likely that tDCS effects reached the OFC as well as other areas related to the aesthetic experience (see Table S1 in [3]). Indeed, primate [40,84,85] and human functional connectivity [86] studies identified extensive anatomically interconnections between the DLPFC and the orbitofrontal cortex, as a great deal of exchange and communication between these areas is essential to plan and execute goal-directed actions [15]. Thus, the major inference that can be drawn from the two reports of Cattaneo and colleagues [10,11] is that interfering with the functioning of the rostro-caudal network involved in aesthetic preference has an effect on the subjective affective response to visual stimuli. Instead, it is unclear whether these effects were related to a DLPFC role in aesthetics or to changes in the OFC’s activity after the DLPFC’s stimulation. Although here I focused mainly on tDCS, homologous concerns can be raised for TMS. For instance, it has been reported that TMS applied on the left (but not right) DLPFC has measurable effects on other prefrontal areas including the medial OFC [87]. Further investigation will be required to broaden our understanding of the neurobiological and neurochemical mechanisms involved in tDCS and TMS to decide about the specific role of the left DLPFC in aesthetic preference as suggested by Cattaneo and colleagues’ results.

## 5. Conclusions

Recent meta-analyses of published neuroimaging studies revealed that multiple and distributed brain regions linked to reward, pleasure, emotion, judgement, decision-making, and perception are involved in aesthetic reactions to artwork [13,88]. Although fMRI studies nearly consistently reported the involvement of the medial OFC in aesthetic appraisal (for absence of activation, see for instance [58,89]), in recent years, some research shifted the focus of our attention towards the dorsolateral section of the PFC. This suggests that an enquiry into the functional characteristics of the left DLPFC with regards to aesthetic appreciation is warranted. This paper argues that, while the OFC is primarily associated with object-appraisal mechanisms (see also [90]), the DLPFC is involved in functions other than the attribution of hedonic value to stimuli.
